# Impact of COVID-19 on Elective Orthopaedic Surgery Outcomes During the Peak of the Pandemic, an Uptick of Complications: An Analysis of the ACS-NSQIP

**DOI:** 10.5435/JAAOSGlobal-D-22-00276

**Published:** 2023-02-20

**Authors:** David Momtaz, Abdullah Ghali, Rishi Gonuguntla, Travis Kotzur, Farhan Ahmad, Andrea Arce, Ariana Olvera, Christina Brady, Ryan Rose

**Affiliations:** From the UT Health San Antonio, San Antonio, TX (Momtaz, Gonuguntla, Kotzur, Arce, Dr. Brady, Dr. Rose); the Baylor College of Medicine, Houston, TX (Dr. Ghali, Olvera); and the Rush University Medical Center, Chicago, IL (Dr. Ahmad).

## Abstract

Since the World Health Organization declared a pandemic in March 2020, COVID-19 has pressured the healthcare system. Elective orthopaedic procedures for American seniors were canceled, delayed, or altered because of lockdown restrictions and public health mandates. We sought to identify differences in the complication rates for elective orthopaedic surgeries before and atfter the pandemic onset. We hypothesized that complications increased in the elderly during the pandemic.

**Methods:**

We conducted a retrospective analysis of the American College of Surgeons–National Surgical Quality Improvement Program database in patients older than 65 years undergoing elective orthopaedic procedures from 2019 (prepandemic) and April to December 2020 (during the pandemic). We recorded readmission rates, revision surgery, and 30-day postoperative complications. In addition, we compared the two groups and adjusted for baseline features with standard multivariate regression.

**Results:**

We included 146,430 elective orthopaedic procedures in patients older than 65 years (94,289 before the pandemic and 52,141 during). Patients during the pandemic had a 5.787 times greater chance of having delayed wait time to the operating room (*P* < 0.001), a 1.204 times greater likelihood of readmission (*P* < 0.001), and a 1.761 times increased chance of delayed hospital stay longer than 5 days (*P* < 0.001) when compared with prepandemic. In addition, during the pandemic, patients were 1.454 times more likely to experience any complication (*P* < 0.001) when compared with patients prepandemic undergoing orthopaedic procedures. Similarly, patients were also 1.439 times more likely to have wound complication (*P* < 0.001), 1.759 times more likely to have any pulmonary complication (*P* < 0.001), 1.511 times more likely to have any cardiac complication (*P* < 0.001), and 1.949 times more likely to have any renal complication (*P* < 0.001).

**Conclusion:**

During the COVID-19 pandemic, elderly patients faced longer wait times within the hospital and increased odds of complications after elective orthopaedic procedures than similar patients before the pandemic.

With the declaration of the COVID-19 pandemic in March 2020, over half the states in the United States recommended the cancellation of all elective procedures.^1^ The mass cancellation affected many elective orthopaedic procedures,^2^ such as carpal tunnel decompressions and total joint arthroplasties, despite their improved outcomes over nonsurgical management.^3^ In addition to these cancellations, the COVID-19 pandemic has been associated with increased wait times to surgery for elective orthopaedic surgeries^4,5^ (*P* < 0.001) and increased length of hospital stay after elective orthopaedic procedures (*P* < 0.001).

Delayed or canceled elective orthopaedic surgeries can result in worse outcomes than procedures that occur in a timely fashion.^6^ Patients experiencing delayed or canceled^7^ procedures can experience longer symptom duration, increased severity, and worse postoperative functional outcomes. Over the coming years, with more literature published, the actual health burden of these delays and cancellations will become increasingly apparent. Elderly patients are increasingly likely to undergo elective orthopaedic surgeries such as arthroplasty to treat degenerative joint changes.^8^ However, this cohort is susceptible to COVID-19 because of relatively weakened immune systems, lower organ function, and increased probability of having multiple underlying diseases.^9^ COVID-19 infection can cause end-organ damage in the lungs, heart, and kidney, as well as hypercoagulable states,^10^ all of which can increase the rates of complications after elective orthopaedic procedures.

Our study uses a multicenter, multi-institutional database to determine whether there is a difference in the rate of postoperative complications after orthopaedic procedures in elderly patients before and during the COVID-19 pandemic. We anticipate that during the pandemic, there was a higher rate of complications after elective orthopaedic procedures in elderly patients.

## Methods

We conducted a retrospective analysis of the American College of Surgeons' National Surgical Quality Improvement Program, a multi-institution and multicenter database that collects patient variables from over 500 hospitals between 2015 and 2020. We included patients older than 65 years undergoing elective orthopaedic procedures in 2019 (prepandemic) and in the last three-quarters of 2020 (during the pandemic).

We collected patient demographic data, American Society of Anesthesiologists' class, smoking history, and comorbidities such as diabetes, congestive heart failure, hypertension, chronic obstructive pulmonary disorder, liver disease with ascites, and dialysis-dependent kidney disease. Body mass index (BMI) was calculated using height and weight. We collected all complications within 30 days: acute renal failure, urinary tract infection, cardiac arrest, myocardial infarction, deep vein thrombosis, pulmonary embolism (PE), pneumonia, cerebrovascular accidents, septic shock, cardiac arrest requiring cardiopulmonary resuscitation, bleeding transfusions, and ascites. We also collected information about wound class, surgical time, discharge destination, length of stay, occurrences of wound disruption, and surgical site infections (superficial and deep). We stratified complications by Clavien-Dindo scores,^11^ with Clavien-Dindo IV scores being considered life-threatening. Complications that were defined as Clavien-Dindo IV were myocardial infarction, occurrences of cardiac arrest requiring cardiopulmonary resuscitation, PE, septic shock, renal failure, and stroke with a neurologic deficit.

### Summary of Demographic Data

A total of 146,430 elective orthopaedic procedures in patients older than 65 years were included, of which 94,289 were included in the prepandemic group, and 52,141 were included in the pandemic group. The mean age of patients was 73 years, with a mean BMI of 30.56 kg/m^2^. Fifty-nine percent of patients were female, 41.0% were male, and <0.01% of patients were nonbinary. In addition, 0.5% of patients were Hispanic, and 5.8% were Black.

### Statistical Analysis

After the exclusion of missing and/or incomplete variables, the sample was split into two cohorts; 2019 before the COVID-19 pandemic and April to December 2020, corresponding to peak cases during the COVID-19 pandemic. The International Business Machines SPSS suite was used to assess and further analyze the data.

Subsequently, an assessment of power was made using the University of California, Los Angeles's Advanced Research Computing Statistical Methods and Data Analysis G*Power Statistics tool. Confidence intervals (CIs) were set at 95%, with statistical significance considered at a *P* value of 0.05.

Next, multivariate regression models were created to control age, BMI, race, and ethnicity. These models were then used to compare the readmission, revision surgery, and 30-day postoperative complication rates between the two cohorts, disclosing significance and CIs. Next, multiple linear and logistic regression models were analyzed for felicitous use to ensure that all requirements were set. Finally, categorical variables were analyzed using chi-square with Kendall's tau or the Fisher exact test when appropriate.

Comparisons between normally distributed data were accomplished with independent sample *t*-tests, and non-normally distributed data were evaluated through the Wilcoxon rank-sum test. Where appropriate, residuals were analyzed for multicollinearity and normal distribution. Furthermore, data were assessed to ensure the selection of proper statistical assessment and that the variables met each test's requirements and assumptions. Continuous data are reported as means with corresponding standard errors and SDs. Categorical results are shown as counts with corresponding column proportions. Odds ratios are provided with their corresponding CIs and *P* values.

## Results

**Figure 1:**
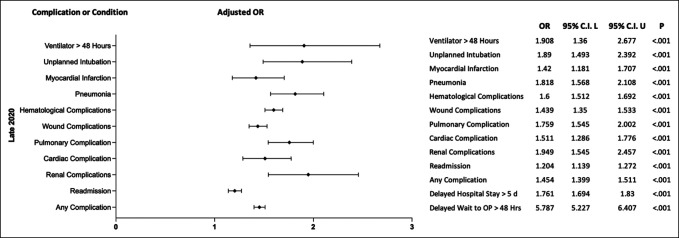
Postoperative adjusted odds ratios of complications for patients undergoing orthopaedic surgery during the pandemic.

**Figure 2:**
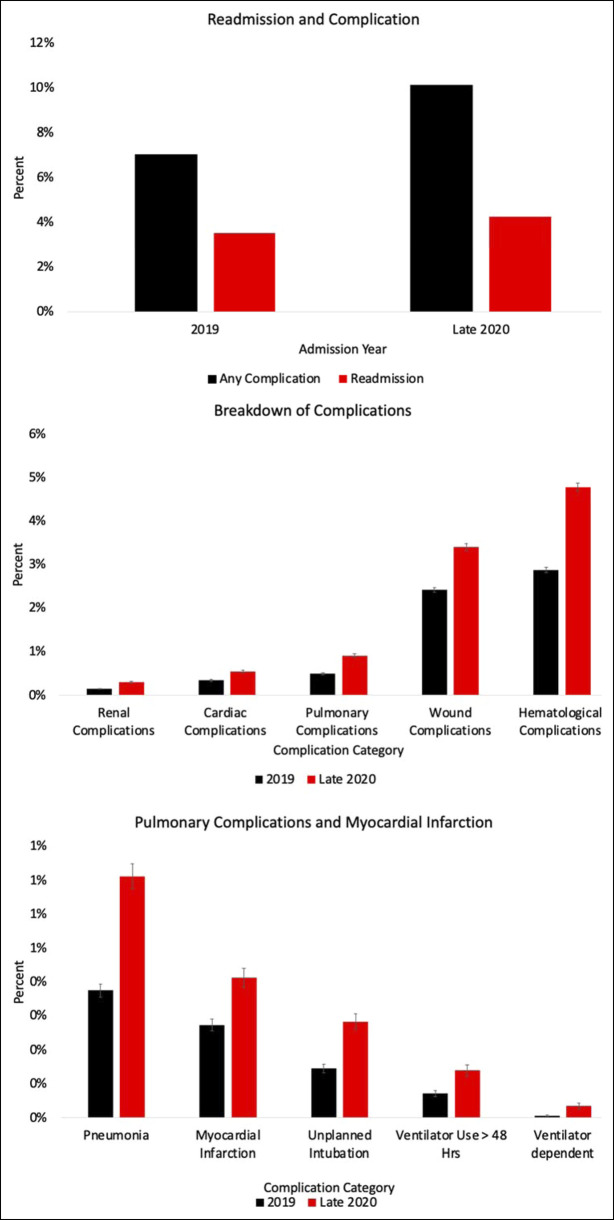
Readmission and complication, breakdown of complications, and pulmonary complications and myocardial infarction are shown in respective breakdowns from top to bottom.

**Figure 3:**
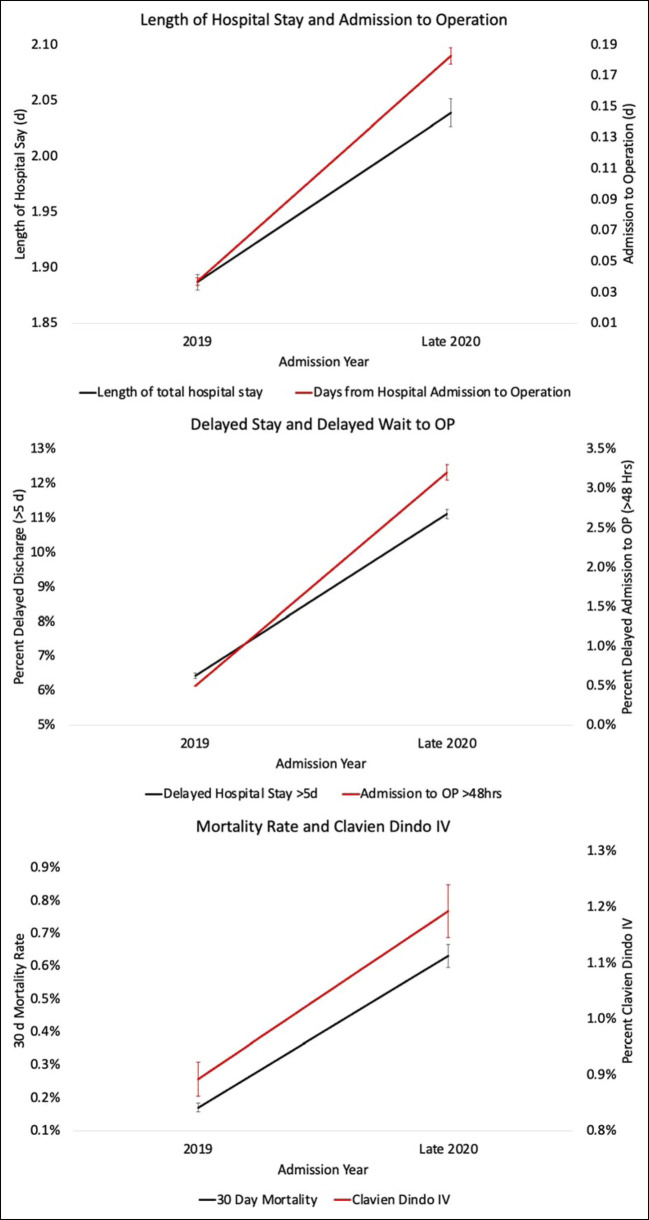
Length of hospital stay and admission to operation, delayed stay and delayed wait to operation, and mortality rate and Clavien-Dindo IV shown from top to bottom.

During the pandemic, patients were 1.454 (95% CI [1.399 to 1.511]; *P* < 0.001) times more likely to experience any complication and 1.439 (95% CI [1.350 to 1.533]; *P* < 0.001) times more likely to have any wound complication when compared with patients undergoing similar procedures before the pandemic (Figure 1 and Figure 2). Similarly, patients were also 1.759 (95% CI [1.545 to 2.457]; *P* < 0.001) times more likely to have any pulmonary complication, 1.511 (95% CI [1.286 to 1.776]; *P* < 0.001) times more likely to have any cardiac complication, and 1.949 (95% CI [1.545 to 2.457]; *P* < 0.001) times more likely to have any renal complication (Figures 1 and2). In addition, patients had a 5.787 (95% CI [5.227 to 6.407]; *P* < 0.001) times greater chance of having delayed wait time to the operating room, a 1.204 (95% CI [1.139 to 1.272]; *P* < 0.001) times greater likelihood of readmission, and a 1.761 (95% CI [1.694 to 1.83]; *P* < 0.001) times increased chance of delayed hospital stay longer than 5 days when comparing patients undergoing elective orthopaedic procedures during the pandemic with before the pandemic (Figure 1 and 3).

## Discussion

Our retrospective review of 146,430 elective orthopaedic procedures found that elderly patients undergoing elective orthopaedic surgeries during the pandemic had higher rates of 48-hour delays between admission and operation and higher rates of delayed hospital stays greater than 5 days. Furthermore, we found that these patients had notably higher rates of 30-day mortality and Clavien-Dindo IV complications than patients undergoing similar procedures before the pandemic. Finally, we found that during the pandemic patients had higher rates of wound, pulmonary, and cardiac complications than those undergoing similar procedures before the pandemic.

Few studies have investigated changes in the rates of complications after elective orthopaedic procedures in elderly patients before and during the pandemic. Unterfrauner et al. studied 5,791 patients from a single institution for 6 months before, 6 months during, and 6 months after the lockdowns. They found that there was no difference in the rates of surgical site infection, wound healing disorders, or noninfectious complications^12^ that coincided with the COVID-19 lockdown. However, their study is much smaller than ours and only includes patients from a single institution. In addition, the burden of COVID-19 on the hospital system in America may be greater than what this institution in Switzerland experienced, which could explain the differences between the findings of our study and their study. Zhong et al. reviewed 40,986 patients in the Premier Healthcare Database of American patients undergoing elective orthopaedic surgeries. They found that during the pandemic, patients were more likely to be readmitted (*P* = 0.006), admitted to the intensive care unit (ICU) (*P* < 0.001), have higher composite complication rates (*P* = 0.002), and have a higher cost of treatment (*P* < 0.001) than patients before the pandemic.^13^ The findings of our study support this, but our study specifically examines elderly patients, a group uniquely affected by the COVID-19 pandemic.

In addition, our study is much larger than the study by Zhong et al. and has greater power because of this larger sample size. Mohammadpour et al.^14^ conducted a prospective cohort study of 6,571 patients undergoing elective and emergent orthopaedic procedures between February 2020 and September 2020. They found a 9.3% mortality rate for patients undergoing these procedures. However, this study did not examine whether there was a difference in the rates of postoperative complications or mortality with the beginning of the COVID-19 pandemic.

The increase in complications after orthopaedic surgeries that coincided with the beginning of the COVID-19 pandemic may be explained by a number of factors. The beginning of the COVID-19 pandemic saw the cancellation of many elective orthopaedic surgeries.

It is possible that patients with more severe conditions were treated during this time. Therefore, they would have had worse postoperative outcomes irrespective of the changes COVID-19 caused in the healthcare landscape. Zagra et al.'s^15^ observational analysis of surgical admissions in Milan found that almost all elective inpatient orthopaedic procedures were canceled over 7 weeks, and only outpatient procedures that could not be postponed were performed.

It is also possible that patients' physical health during the pandemic worsened. Stockwell et al.'s^16^ review of 66 studies and 86,981 participants found a decrease in physical activity levels in adults that corresponded to the beginning of the COVID-19 pandemic. In addition, Oliveira et al. conducted a systematic review of 25 studies. They found that the beginning of the COVID-19 pandemic corresponded to a notable decline in the physical activity levels of elderly patients and increased sitting time in these same patients. Increased physical activity decreases the risk of chronic disease, and patients with fewer chronic diseases intuitively experience better outcomes after operation. Therefore, the decrease in physical activity in elderly patients during the COVID-19 pandemic should be considered when evaluating patients' risk to mitigate postoperative complications. This may have also restricted nonsurgical treatment such as physical or occupational therapy and worsening symptoms.

The cancellation of elective orthopaedic procedures has had other negative impacts on patients. Delays and cancellations of operations increase the time that patients experience pain, decreased mobility, and worsened quality of life.^17,18^ Few studies have investigated the effect of cancellations on patient quality of life during the pandemic. Knebel et al.'s^19^ study of 77 patients found that cancellation of elective orthopaedic procedures was associated with an increase in the level of self-reported pain (*P* < 0.0001) despite increased analgesic use and, in female patients, a self-reported increase in depressive feelings (*P* = 0.046) when compared with when the surgery was scheduled. Patients during the COVID-19 pandemic who experienced cancellations and delays were subject to these adverse outcomes and should be appropriately treated for these complications.

## Limitations

Although the large size of the American College of Surgeons' National Surgical Quality Improvement Program database is a massive strength of this study, it also introduces some limitations. The retrospective nature of this study presents an inherent bias to our findings. In addition, our investigation is limited by the data collected by the database. The database contains no patient-reported outcomes, which limits our ability to determine postoperative outcomes. In addition, no radiographs or detailed histories are available, which limits our ability to determine whether there was a difference in the severity of the patients being treated during the pandemic and before the pandemic. Furthermore, no details about hospital bed availability, surgical approach, rehabilitation, and length of the total follow-up were included in the database. These are all factors that may have influenced postoperative outcomes, and the deficiency of these limits our findings.

## Conclusion

The COVID-19 pandemic in March 2020 caused the US healthcare system to undergo several changes. One of these changes was a mass cancellation of elective orthopaedic surgeries. Because of the well-documented relationship between adverse outcomes after orthopaedic procedures and old age, physicians must be aware of the effects these phenomena have when managing the care of elderly patients. Our retrospective analysis found that during the COVID-19 pandemic, elderly patients faced longer hospital wait times and increased odds of complications after elective orthopaedic procedures than similar patients before the pandemic.
